# Slow-wave modulation analysis during states of unconsciousness using the novel tau-modulation method

**DOI:** 10.1088/1741-2552/ace5db

**Published:** 2023-07-21

**Authors:** Tao Xie, Zehan Wu, Thomas J Foutz, Xinjun Sheng, Xiangyang Zhu, Eric C Leuthardt, Jon T Willie, Liang Chen, Peter Brunner

**Affiliations:** 1 Department of Neurosurgery, Washington University School of Medicine, St. Louis, MO, United States of America; 2 National Center for Adaptive Neurotechnologies, St. Louis, MO, United States of America; 3 Department of Neurosurgery, Huashan Hospital, Fudan University, Shanghai, People’s Republic of China; 4 Department of Neurology, Washington University School of Medicine, St. Louis, MO, United States of America; 5 State Key Laboratory of Mechanical System and Vibration, Shanghai Jiao Tong University, Shanghai, People’s Republic of China; 6 Department of Neurology, Albany Medical College, Albany, NY, United States of America

**Keywords:** slow-wave, modulation, phase-amplitude coupling, unconsciousness, cross-correlation, broadband gamma, electrocorticography

## Abstract

*Objective*. Slow-wave modulation occurs during states of unconsciousness and is a large-scale indicator of underlying brain states. Conventional methods typically characterize these large-scale dynamics by assuming that slow-wave activity is sinusoidal with a stationary frequency. However, slow-wave activity typically has an irregular waveform shape with a non-stationary frequency, causing these methods to be highly unpredictable and inaccurate. To address these limitations, we developed a novel method using tau-modulation, which is more robust than conventional methods in estimating the modulation of slow-wave activity and does not require assumptions on the shape or stationarity of the underlying waveform. *Approach*. We propose a novel method to estimate modulatory effects on slow-wave activity. Tau-modulation curves are constructed from cross-correlation between slow-wave and high-frequency activity. The resultant curves capture several aspects of modulation, including attenuation or enhancement of slow-wave activity, the temporal synchrony between slow-wave and high-frequency activity, and the rate at which the overall brain activity oscillates between states. *Main results*. The method’s performance was tested on an open electrocorticographic dataset from two monkeys that were recorded during propofol-induced anesthesia, with electrodes implanted over the left hemispheres. We found a robust propagation of slow-wave modulation along the anterior–posterior axis of the lateral aspect of the cortex. This propagation preferentially originated from the anterior superior temporal cortex and anterior cingulate gyrus. We also found the modulation frequency and polarity to track the stages of anesthesia. The algorithm performed well, even with non-sinusoidal activity and in the presence of real-world noise. *Significance*. The novel method provides new insight into several aspects of slow-wave modulation that have been previously difficult to evaluate across several brain states. This ability to better characterize slow-wave modulation, without spurious correlations induced by non-sinusoidal signals, may lead to robust and physiologically-plausible diagnostic tools for monitoring brain functions during states of unconsciousness.

## 1. Introduction

States of unconsciousness (e.g. anesthesia, non-rapid eye movement, sleep, and coma) are often characterized by their distinct electrophysiological signatures. One feature that is common to all of them is slow-wave activity, seen electrographically as large deflections alternating at a rate of approximately 1 Hz. These waves are thought to be large-scale indicators of underlying brain states in which neurons alternate between sustained periods of depolarization (up-states) and hyperpolarization (down-states). Neurons tend to repetitively fire in the depolarized up-state, and remain silent for several hundred milliseconds in the hyperpolarized down-state (Steriade *et al* 1993, Massimini *et al* 2004, Murphy *et al* 2009, 2011).

Slow-waves have been extensively studied during non-rapid eye movement sleep (Massimini *et al* 2004, Murphy *et al* 2009) and under anesthesia (Murphy *et al* 2011) to investigate the complexity of the underlying network interactions. Slow-wave activity is initiated, maintained, and terminated through the interplay between intrinsic network interactions, and modulated by different factors, such as excitability levels (Sancristóbal *et al* 2016), neuromodulators (Pigorini *et al* 2015, D’Andola *et al* 2018, Nghiem *et al* 2020), and inputs from other connected areas (Sanchez-Vives 2020). Slow-waves have been suggested as the default emergent activity of the cortical network (Sanchez-Vives and Mattia 2014, Sanchez-Vives *et al* 2017, Sanchez-Vives 2020).

Several studies have examined how the temporal and spatial distribution of slow-wave dynamics relate to the states of unconsciousness (Purdon *et al* 2015, Brown *et al* 2018, Kremen *et al* 2019, von Ellenrieder *et al* 2022). During propofol-induced anesthesia, the onset of slow-wave is correlated with loss of consciousness (LOC) (Cimenser *et al* 2011, Lewis *et al* 2012). Furthermore, there has been particular interest in the modulation of the amplitude of high-frequency neural activity by the phase of low-frequency oscillations (i.e. phase-amplitude coupling, Tort *et al* 2010). Recent evidence suggests a functional role of this modulation during LOC (Purdon *et al* 2013, Mukamel *et al* 2014, Huang *et al* 2018, Malekmohammadi *et al* 2019, Stephen *et al* 2020, Dong *et al* 2022, Liu *et al* 2022). Specifically, the slow-wave modulated alpha oscillations at different phases (trough-max and peak-max), depending on the depth of anesthesia (Purdon *et al* 2013, Mukamel *et al* 2014). Further, slow-wave modulated broadband signal across different regions, reflecting underlying cortical up- and down-states of unconsciousness (Stephen *et al* 2020).

Conventional slow-wave modulation analyses are based on phase analysis after narrow-band filtering, which may be confounded by sharp signal transitions and nonsinusoidal waveforms (Aru *et al* 2015, Cole and Voytek 2017). In these approaches, slow-wave modulation is extracted in three steps: (1) narrow-band bandpass filtering of the neuronal activity into slow-wave and high-frequency band signals; (2) extracting the phase of the slow-wave signal and the power of the high-frequency signal; and (3) quantifying the correlation between the phase and the power (Canolty *et al* 2006, Tort *et al* 2010, Purdon *et al* 2013). These approaches assume that slow-wave activity can be well-characterized by sinusoidal waveforms. However, this is an inappropriate assumption, as slow-wave activity often has an irregular waveform shape and frequency (Stephen *et al* 2020). Because of this, conventional methods that use narrow-band filtering to extract the phase of the slow-wave activity are highly susceptible to producing spurious coupling in the presence of non-sinusoidal waveforms, leading to an unreliable representation of slow-wave modulation (Cole and Voytek 2017).

Slow-wave activity can be characterized by three general features: modulation strength, polarity, and frequency. Together they may represent different neurophysiological aspects under the state of unconsciousness. For example, modulation strength represents the attenuation or enhancement of slow-wave activity, modulation polarity represents the temporal synchrony between slow-wave and high-frequency activity, and modulation frequency represents the rate at which the activity fluctuates between up and down states. Prior studies have primarily focused on the modulation strength and polarity (Massimini *et al* 2004, Purdon *et al* 2013, 2015). However, conventional slow-wave modulation methods assume a stationary modulation frequency, making it difficult to accurately extract the modulation frequency of slow-wave activity that is characterized by instantaneous and non-stationary neural activities.

**Figure 1.**
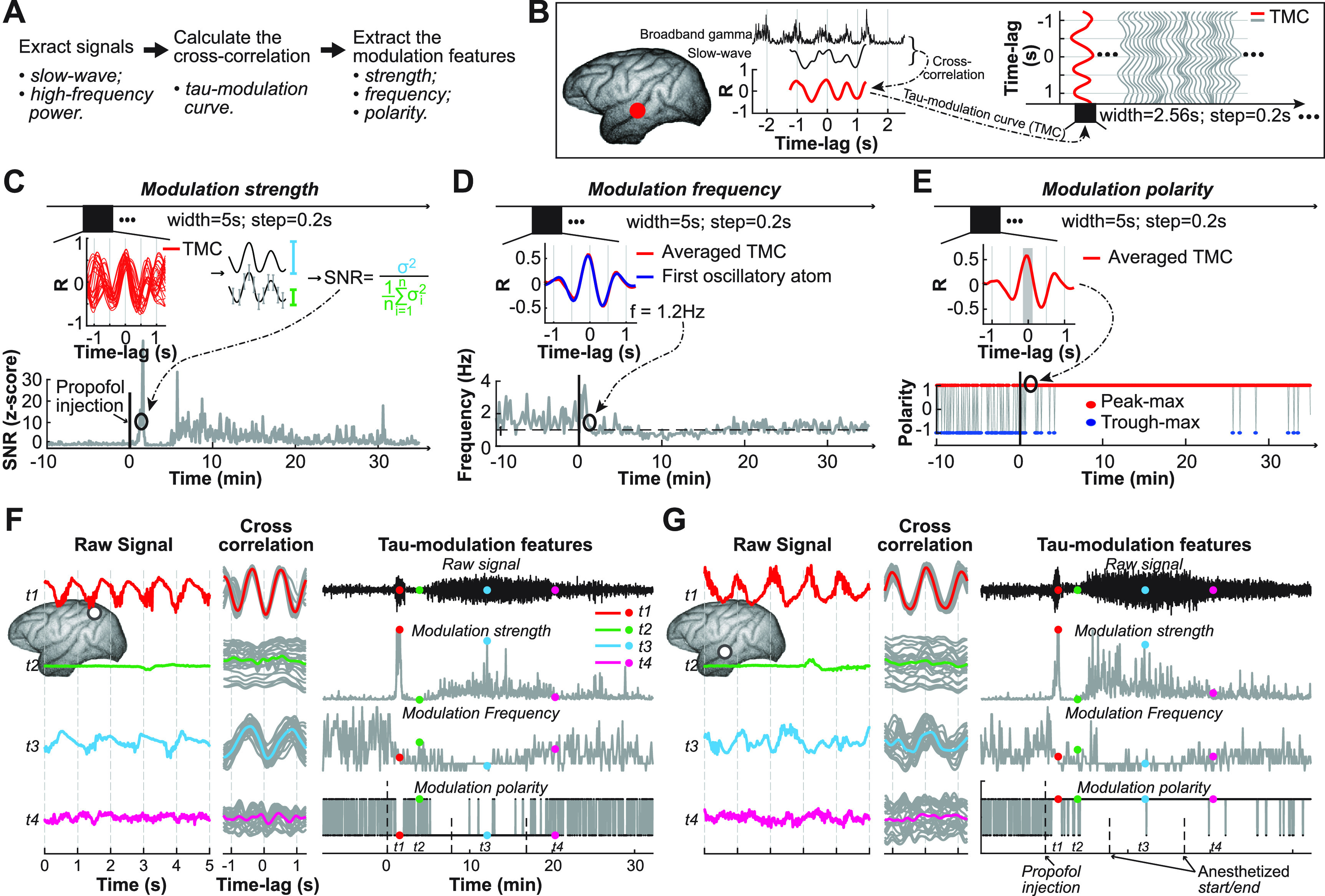
Method description. (A) General steps of tau-modulation method. (B) The tau-modulation curve (TMC) is defined as the cross-correlation coefficient values (*R*) between the slow-wave and broadband gamma power. (C)–(E) Extracting the three modulation features from the tau-modulation curve. (C) Calculating the signal-to-noise ratio (SNR, Schalk *et al* 2007) of TMCs within a 5 s-long observation window; (D) averaging the TMCs within a 5 s-long observation window and applying a matching pursuit algorithm (Chandran *et al* 2016) to extract modulation frequency; (E) averaging the TMCs within a 5 s-long observation window, removing the linear trend, and determining the positive or negative polarity of the averaged TMC around time-lag 0. These steps were repeated every 0.2 s. (F), (G) Applying the tau-modulation method to the signals recorded from two representative electrodes. The left panel shows the raw signal within four different periods (t1–t4); the middle panel shows the cross-correlation (i.e. tau-modulation curve) between the slow-wave and broadband gamma activity; the right panel depicts the raw signal and three modulation features extracted from the tau-modulation curve.

To address these limitations of conventional slow-wave modulation extraction methods, we describe a novel method (tau-modulation) to estimate slow-wave modulation (figure 1). This method determines a tau-modulation curve (TMC) using a cross-correlation between the slow-wave and the instantaneous power of the band-pass filtered high-frequency signal. It then extracts three modulation features (i.e. strength, frequency, and polarity) that represent different neurophysiological signatures under the state of unconsciousness. In contrast to conventional methods, tau-modulation does not depend on estimating the phase of the slow-wave, making it less prone to spurious coupling in the presence of non-sinusoidal waveforms. Additionally, tau-modulation extracts the instantaneous modulation frequency as well as the modulation strength and polarity, enabling it to conduct more comprehensive monitoring of different states of unconsciousness. This improved performance and robustness against artifacts could help improve the efficiency and reliability of slow-wave modulation methods and could lead to better diagnostic tools for monitoring brain functions during states of unconsciousness.

## 2. Material and methods

### 2.1. Data collection

We used propofol-induced anesthesia datasets to verify the tau-modulation method. The datasets were recorded at the RIKEN Institute (Japan) and are publicly available as part of the Neurotycho database (http://neurotycho.org/anesthesia-task). The protocol was approved by the RIKEN Ethics Committee (Yanagawa *et al* 2013). Two macaca fuscata monkeys (Chibi, C; George, G; supplementary figure 1) took part in the propofol-induced anesthesia study, and each monkey repeated the experiment on two separate days (C1, C2, G1, G2).

Electrocorticography (ECoG) data were recorded with a sampling frequency of 1 kHz using chronically implanted multichannel electrode arrays (Unique Medical, Japan). The arrays, consisting of 128 platinum electrodes spaced at 5 mm inter-electrode distance, were implanted in the subdural space and covered the majority of the lateral surface of the left hemisphere and the medial cortical regions. Reference electrodes were made of rectangular platinum plates placed within the subdural space between the ECoG array and dura. Ground electrodes were placed within the epidural space.

Throughout the experiments, the monkey was seated in a primate chair with both arms and head movement restricted. The experiment was comprised of three conditions: (1) awake; (2) anesthetized; and (3) recovery. The awake condition had periods with eyes-uncovered and eyes-covered. First, the monkey had its eyes uncovered and sat at rest for up to 20 min. Next, the monkey’s eyes were covered by an eye mask to refrain from evoking visual responses. During the eyes-covered period, the monkey sat at rest for up to 20 min before propofol (5.2 mg kg^−1^ for C1 and C2, 5.0 mg kg^−1^ for G1 and G2) was injected intravenously. LOC was defined as the point at which the monkey no longer responded to manipulation of the monkey’s hand or touching of the nostril or philtrum with a cotton swab. As an additional confirmation that the monkey had reached LOC, slow-wave activities were observed in the neural signal. Following the LOC, the monkey was left anesthetized to recover on its own from anesthesia (recovery condition). Throughout the recovery condition, there were eyes-covered and eyes-uncovered periods. During the recovery period, the monkey’s eyes were covered, and the monkey sat calmly for 10–30 min. Next, the monkey’s eyes were uncovered, and the monkey sat at rest for about 10 min. The onset of recovery was defined as the point at which the monkey started to respond to manipulation of the monkey’s hand or touching of the nostril or philtrum with a cotton swab.

Data processing was performed using EEGLAB functions (Delorme and Makeig 2004) and custom scripts written in MATLAB (Mathworks, Inc.). We pre-processed the signals using a common average reference spatial filter to remove common noise across all electrodes (Liu *et al* 2015), and two finite impulse response (FIR) notch filters (48–52 Hz, 98–102 Hz) to remove line noise.

### 2.2. Tau-modulation method

The tau-modulation method is comprised of three principle steps (figure 1(A)): (1) extracting slow-wave and high-frequency power; (2) calculating the cross-correlation between slow-wave and high-frequency power along each time-lag (*τ* or tau), resulting in a series of correlation coefficients (i.e. TMC); and (3) extracting the three features (strength, frequency, polarity) from the TMC. This method was applied to the signal from each of the electrodes.

Slow-wave activity reflects a rhythmic alteration between up- and down-states. Power spectral changes of ECoG signals recorded in the broadband gamma range correlate with population-level activity and represent underlying local multi-unit spiking activity (Nir *et al* 2007, Ray *et al* 2008, Manning *et al* 2009, Miller *et al* 2014). Thus, broadband gamma activity can reliably reflect neuronal activation during up-state and distinguish it from down-state periods (Stephen *et al* 2020). This evidence presents the primary motivation for applying the tau-modulation analysis to determine the coupling between slow-wave and broadband gamma activity. Prior studies (Miller *et al* 2009, 2014) showed that broadband gamma is a spectrally broad but spatially focal, non-rhythmic neural activity that occurs above ∼40 Hz. In our analysis, we chose the broadband gamma range as the 55–145 Hz band, to exclude the influence of line noise at 50 Hz and its harmonic at 150 Hz. The MATLAB code that implements the tau-modulation methods and sample data is openly available on GitHub (https://github.com/neurotechcenter/TauModulation_SlowWave).

#### 2.2.1. Extracting TMC

We first extracted slow-wave (0.2–4 Hz) and broadband gamma activity (55–145 Hz) using FIR bandpass filters ($pop\_eegfiltnew()$, EEGLAB). We then applied a Hilbert transform on the broadband gamma activity to extract its envelope. Finally, we down-sampled ($pop\_resample()$, EEGLAB) the resulting signals, i.e. the slow-wave activity and broadband gamma envelope, to 25 Hz to reduce the computation complexity of our analysis. Next, we calculated the cross-correlation between the slow-wave and broadband gamma envelope. Specifically, we calculated the Pearson correlation between the 2.56 s-long Hamming-window-filtered slow-wave segment and the broadband gamma envelope segment with a time-lag ranging from −1.28 s to 1.28 s, which yielded one TMC (red line in figure 1(B)). We repeated this procedure every 0.2 s to yield a series of TMCs along time (grey line in figure 1(B)). Note that this analysis did not involve estimating the phase of the slow-wave, a typical step employed by conventional slow-wave modulation analysis methods, which makes our tau-modulation method less prone to spurious correlation induced by non-sinusoidal waveforms. Finally, we extracted the three modulation features (i.e. strength, frequency, and polarity) from the TMCs as described below.

#### 2.2.2. Extracting the modulation strength

We calculated the signal-to-noise ratio (SNR) of TMCs within a 5 s-long observation window (i.e. 25 TMCs over 5 s). Specifically, we calculated the SNR as the ratio between the variance of the whole TMC period and the average variance of the individual sample during the TMC period (figure 1(C), equation (1), Schalk *et al* (2007)). SNR values larger than 1 indicate that broadband gamma activity was modulated by the slow wave, while SNR values close to 1 indicate no modulation. This procedure was repeated every 0.2 s to yield a series of SNR values over time. We chose the awake eyes-covered period as the baseline. We normalized the SNR of each electrode by subtracting the average SNR of the baseline and dividing it by the standard deviation of the baseline. The SNR values tended to be increased after propofol injection. The SNR was calculated as follows:

\begin{align*} \textrm {SNR} = \frac{\sigma^2}{\frac{1}{n}\sum_{i = 1}^{n}\sigma^2_{i}}. \end{align*}

In this equation, *σ* ^2^ (blue error bar in figure 1(C)) indicates the variance of the *R* values across whole time-lags (red traces in figure 1(C)). $\sigma_i^2$ (grey error bar in figure 1(C)) indicates the variance of the *R* values during each time-lag. *n* indicates the number of time-lags (i.e. 64; calculated as the feature rate of 25 samples per second × the 2.56 s length of the whole time-lags).

To determine the peak in the tau-modulation strength (figure 3(C)), we first approximated the electrode’s normalized SNR values over the 5–20 min after propofol injection using a 20th-order polynomial curve (supplementary figure 3(B)). This approximation created a temporally smoothed representation of the tau-modulation strength and allowed us to determine the point in time where the modulation strength of each electrode reached its first maximum (green dots, figure 3(C), supplementary figure 3). Specifically, we chose the first peak of the fitted line that exceeded the average SNR values within the anesthetized period.

#### 2.2.3. Extracting the modulation frequency

We averaged the TMCs within a 5 s-long observation window and applied a matching pursuit algorithm (Chandran *et al* 2016) to extract the fundamental oscillation frequency of the averaged TMC. Matching pursuit is a technique that iteratively approximates the original signal using a linear combination of Gabor atoms, enabling it to capture both sharp transient and sustained oscillatory elements. We defined the first oscillatory atom as the modulation frequency. This procedure was repeated every 0.2 s to yield a series of modulation frequency values along time. We found the modulation frequency to be approximately 1 Hz after propofol injection (grey line, figure 1(D)).

#### 2.2.4. Extracting the modulation polarity

The modulation polarity is the sign of the cross-correlation between slow-wave and broadband gamma envelope. At any given time-lag of the cross-correlation, if the sign is positive, it indicates that the slow-wave and broadband gamma are positively coupled and thus increase and decrease together (peak-max); if the correlation is negative, it means that the slow-wave and broadband gamma are negatively-coupled and thus increase when the other decreases (trough-max). To calculate the modulation polarity, we averaged the TMCs within a 5 s-long observation window and removed the linear trend of the resulting averaged TMC. We then analyzed the correlation between slow-wave and broadband gamma. We found that under propofol anesthesia, broadband gamma was much more likely to couple to the peak or the trough of the slow-wave than to the rising or falling phases (supplementary figure 7, Purdon *et al* 2013). Hence a positive/negative value around a time-lag of 0 represents that broadband gamma amplitude is higher during the peak/trough of the slow-wave (peak-max/trough-max). For example, figure 1(E) shows an averaged TMC with a positive value around time-lag 0 (grey shaded area), representing a peak-max phenomenon during the 5 s period. This procedure was repeated every 0.2 s and yielded a series of modulation polarity values along time (1: peak-max, red cycle; -1: trough-max, blue cycle). This specific electrode shown in figure 1(E) exhibited a consistent peak-max slow-wave modulation after propofol injection.

## 3. Results

### 3.1. Electrophysiological effects of propofol

**Figure 2.**
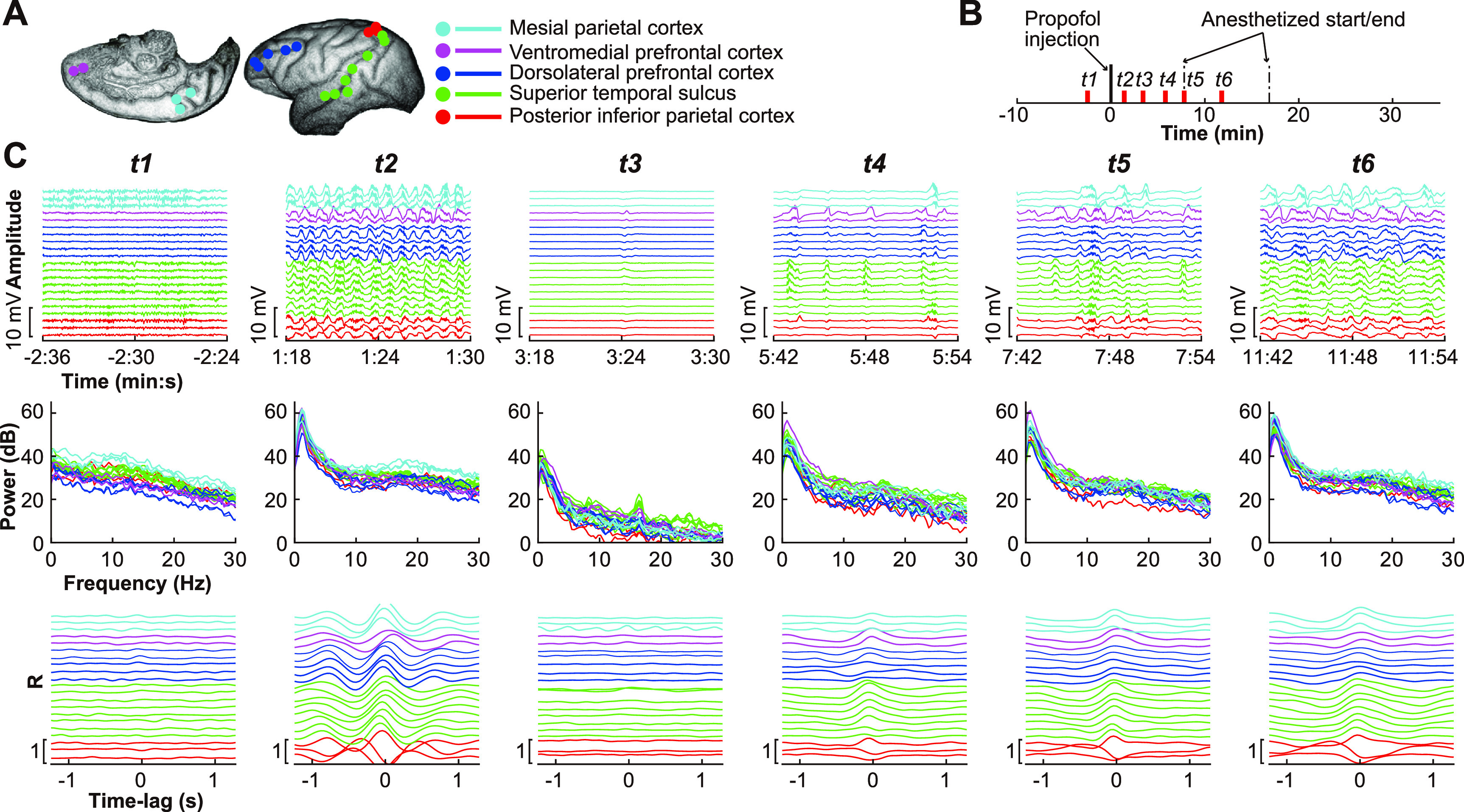
Electrophysiological effects of propofol. (A) Representative electrode locations within five distinct functional cortical areas. We chose 21 electrodes within five functionally relevant cortical areas to illustrate the effect of propofol on the monkey’s cortex. (B) Timeline of the propofol anesthesia procedure. We chose six pivotal periods (t1–t6) to illustrate the neural signal dynamics before and after propofol injection. (C) Detailed signal properties during the six time periods (signals for the same electrodes as in (A). The upper panel shows raw signals, the middle panel shows the spectral power, and the lower panel shows the tau-modulation curves.

To illustrate how tau-modulation characterizes the electrophysiological dynamics during propofol anesthesia, we selected signals from temporal, parietal, and prefrontal cortex in monkey Chibi (C1, figure 2(A)) at six time periods (t1–t6, figure 2(B)). The upper panel shows raw signals (figure 2(C)). Prior to propofol injection, the ECoG signal exhibits a wide range of neural activity (t1). Within one minute of propofol injection, the ECoG signal exhibits synchronized slow-wave activity for up to one minute (t2). This is followed by a general suppression of neural activity for several minutes (t3) and a steady increase of slow-wave activity over the next 10 min (t4–t6). The middle panel shows the spectral power. The neural signals exhibit a typical 1/f spectrum during baseline (t1). Spectral power increases within one minute of propofol injection, especially in slow-wave frequencies (t2). This is followed by a general decrease in spectral power (t3). Spectral power remains suppressed, except for an increase in low-frequency power, indicating an increased slow-wave activity (t4–t6). The lower panel shows the TMC, which characterizes the slow-wave modulation activity following the propofol injection (t2) and the period during which slow-wave activity returns (t4–t6).

### 3.2. Modulation strength

**Figure 3.**
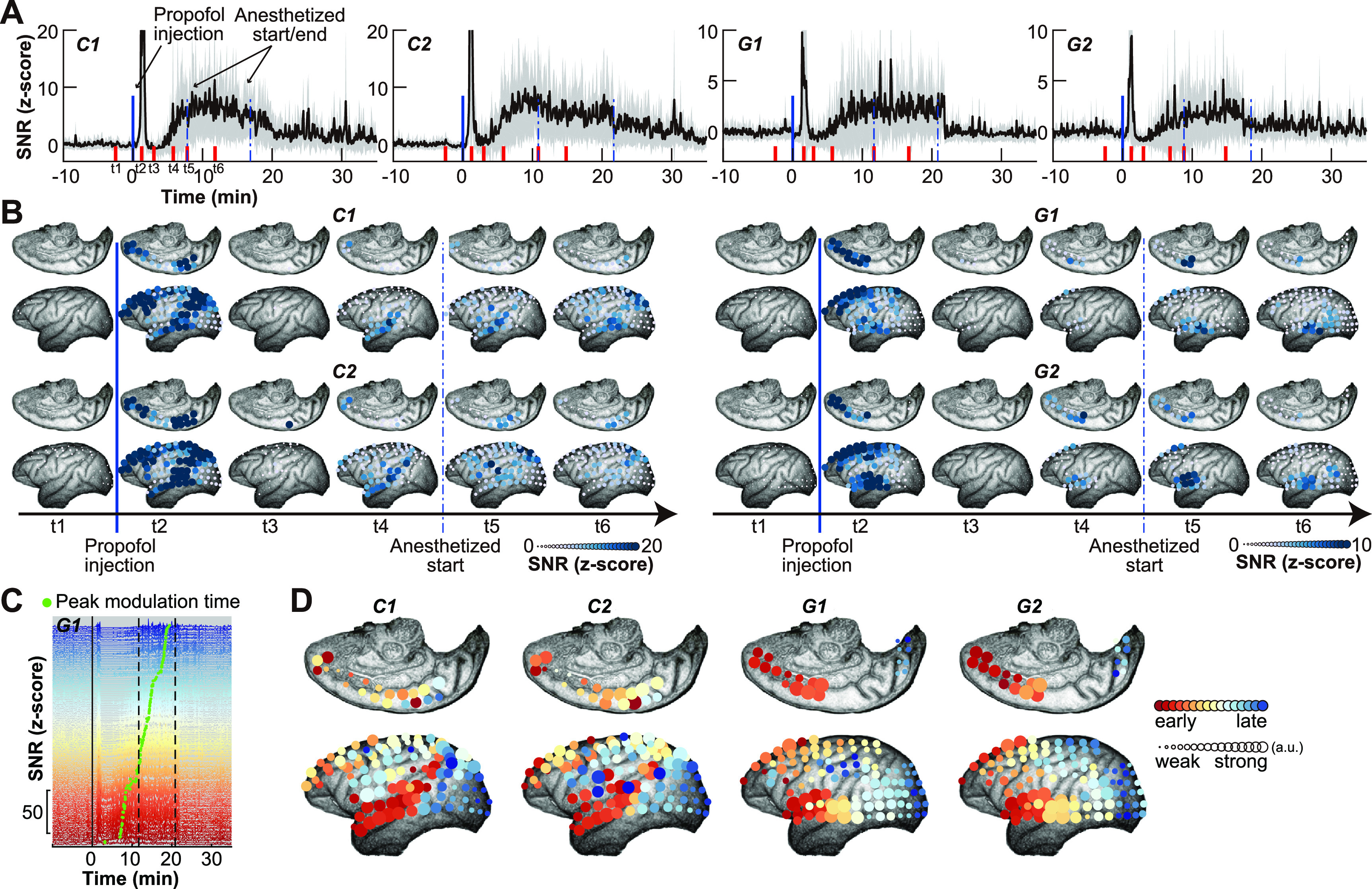
Spatial-temporal dynamics of tau-modulation strength. (A) Averaged (mean±s.d., *n* = 128) tau-modulation strength values across all electrodes for each dataset (C1, C2, G1, G2). (B) Tau-modulation strength of individual electrode within six time periods (t1–t6, red bar in A). (C) Tau-modulation curves for each electrode of dataset C1, which are sorted by the peak modulation time (green dots). (D) Propagation of tau-modulation strength across the entire cortex (color represents the peak modulation time, and circle size represents the corresponding modulation strength).

To understand how slow-wave modulation characterizes changes in the electrophysiological activity after propofol injection, we averaged the tau-modulation strength values across all the electrodes (figure 3(A)). We found a sudden sharp increase of the modulation strength within one minute of propofol injection, followed by a return to baseline and a gradual increase within the following minutes. This increase faded away within the next half hour. This evolution of the neural activity is indicated in figure 2.

To investigate the spatial-temporal dynamics of slow-wave modulation, we analyzed the tau-modulation strength of individual electrodes (figure 3(B)) within six time periods (t1–t6, red bar in figure 3(A)). The results indicate the dynamic networks evolved in two distinctly different stages. The first stage (around t2) encompasses a roughly one-minute-long period following propofol injection. The spatial dynamics of this period resemble those of the default mode networks (supplementary figure 2) known to have vast anatomical connections throughout the brain, and are considered to relate to high-order functions (Binder *et al* 2009, Buckner and DiNicola 2019).

The second stage (t3–t6) encompasses roughly 5–20 min after propofol injection. During this stage, spatiotemporal dynamics of the tau-modulation strength indicate the propagation of the effect of propofol on the individual brain regions. We analyzed tau-modulation strength across the cortex throughout the second stage to determine whether there is a clear propagation effect with early peak tau-modulation strength across time and electrode locations (green dots, figure 3(C), supplementary figure 3). We found that the propagation of modulation strength (figure 3(D)) traveled across the entire cortex (color represents the peak modulation time, and cycle size represents the corresponding modulation strength). Our results show the spatiotemporal propagation along the anterior–posterior axis of the lateral aspect of the cortex. We further demonstrate that this propagation preferentially originated from the anterior superior temporal cortex and anterior cingulate gyrus (supplementary figure 4).

The tau-modulation method was compared to conventional methods for characterizing modulation, including the modulation index as described in Canolty *et al* (2006). This comparison found that the results generally aligned with one another, and tau-modulation demonstrated a stronger modulation effect, indicating a higher sensitivity and robustness in monitoring brain states (supplementary figure 5). Additionally, the interpretation of slow-wave as cortical up-state and down-state suggests that neural activity at all frequencies is coupled to slow-wave activity. This is supported by findings that up- and down-states equally affect rhythmic and non-rhythmic neural activities (Lewis *et al* 2012). We further confirmed this phenomenon by determining the effect of slow-wave modulation on different frequency bands (i.e. theta, alpha, beta, and low gamma), which demonstrated similar findings (supplementary figure 6).

### 3.3. Modulation frequency

**Figure 4.**
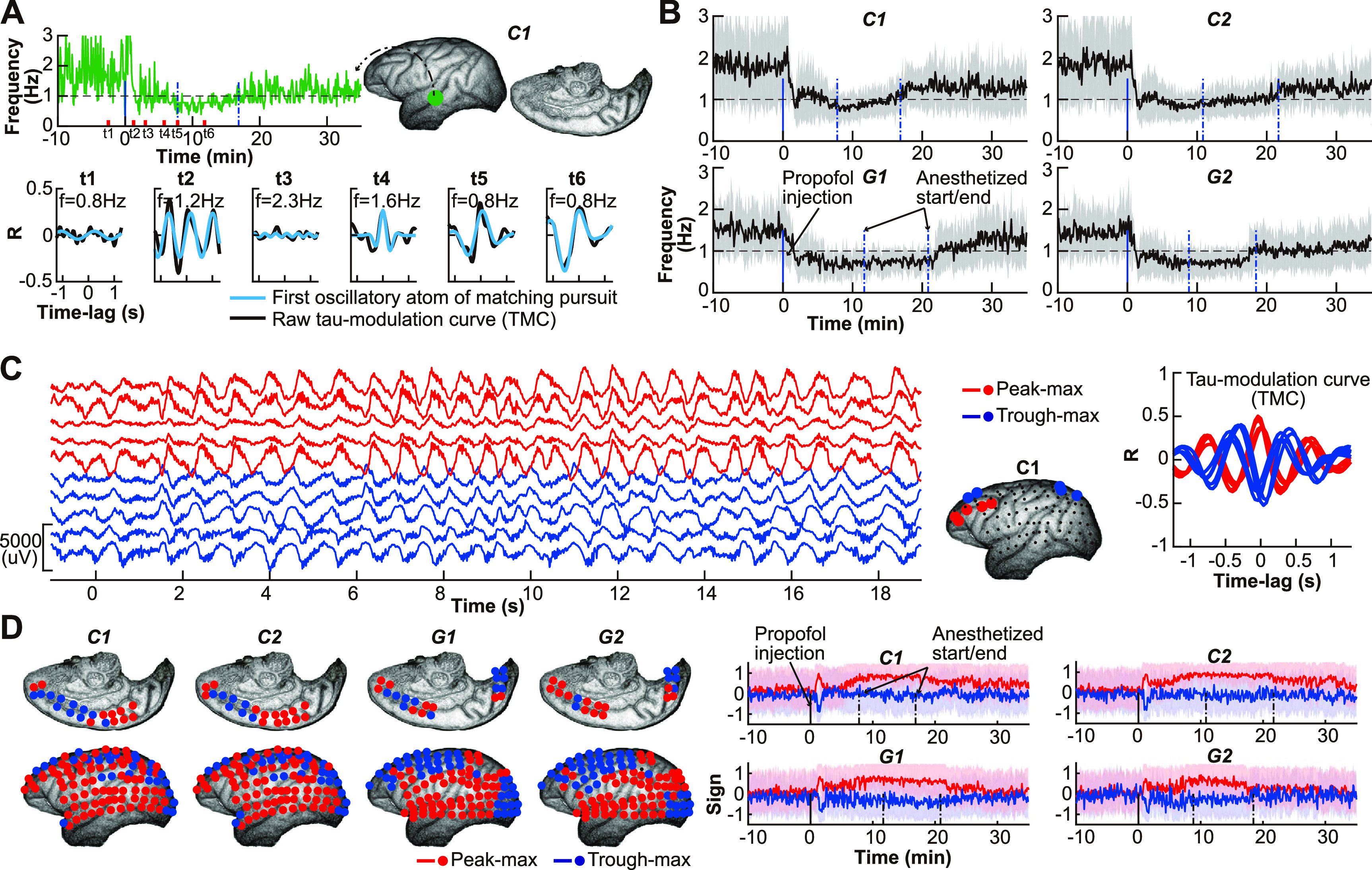
Tau-modulation frequency and polarity. (A) Upper panel shows the modulation frequency for one electrode near the superior temporal sulcus in dataset C1. The modulation frequency was defined as the first oscillatory atom from the matching pursuit algorithm (Chandran *et al* 2016). The lower panel shows the tau-modulation curve (black) and the first oscillatory atom (cyan) during six time periods (t1–t6, red bar in upper panel). These results indicated that the matching pursuit algorithm could accurately extract the modulation frequency. (B) Averaged (mean±s.d., *n* = 128) modulation frequency across all electrodes for each dataset (C1, C2, G1, G2). (C) ECoG signals (left panel) and the corresponding tau-modulation curve (right panel) with opposite modulation polarity. Red and blue indicate peak-max and trough-max, respectively. (D) Left panel: average modulation polarity values around the sharp modulation period (roughly one minute following propofol injection). Electrodes with positive/negative averaged polarity values were indicated as peak-max/trough-max modulation electrodes (red/blue). Right panel: averaged (mean±s.d.) modulation polarity for peak-max/trough-max modulation electrodes (red/blue).

We used a matching pursuit algorithm (figure 4(A)) to extract the modulation frequency from the TMC that corresponds to the specific frequency of slow-wave modulation due to propofol anesthesia. We then averaged the tau-modulation frequencies across all electrodes (figure 4(B)). Similar to the modulation strength, we found a sudden sharp transition in the modulation frequency from a wide range of frequencies to converge at a single frequency around 1 Hz. This transition occurred within one minute of propofol injection, followed by a short period of instability, and then a gradual upward trend in modulation frequency over the following minutes.

### 3.4. Modulation polarity

We found that a positive/negative modulation polarity around time-lag 0 (figure 4(C)-right) could faithfully reflect the peak-max/trough-max modulation (figure 4(C)-left). We averaged the tau-modulation polarity values across the peak-max and trough-max modulation electrodes (figure 4(D)-left), and found a separation of modulation polarity towards the end of the anesthesia (figure 4(D)-right).

## 4. Discussion

The tau-modulation method is a novel approach that provides insight into slow-wave modulation and propagation during states of unconsciousness. We demonstrated that this method could extract modulation strength, frequency, and polarity from a simple TMC derived from the cross-correlation between the slow-wave and high-frequency signals. Unlike conventional methods, tau-modulation does not require an estimation of the phase of slow-wave activity, making it less prone to the confounding effects induced by non-sinusoidal brain waves. We validated this method by applying it to electrocorticographic signals recorded during propofol-induced anesthesia and showed that the results were comparable to conventional methods.

Our results showed a strong slow-wave modulation strength over the default mode network (supplementary figure 2). Positron emission tomography and functional magnetic resonance imaging studies found multiple cortical areas that were maximally involved in slow-wave activity, including the left inferior frontal gyrus, the entire extent of the cingulate gyrus (from anterior to posterior), the precuneus and the insula (Maquet 2000, Dang-Vu *et al* 2005, Kaufmann *et al* 2006, Riedner *et al* 2011). EEG studies also showed similar areas of involvement (Massimini *et al* 2004, Murphy *et al* 2009, 2011). The overlap between the default mode network and the areas maximally involved by slow-wave activity highly suggests a neurophysiologic association (Huber *et al* 2004, 2006, Van Someren *et al* 2011).

Slow-waves can have distinct cortical origins. For example, surface EEG analyses have suggested that the transition from down-state to up-state originates in the lateral anterior-most part of the scalp (Massimini *et al* 2004). Source modeling of high-density EEG has found diffuse hotspot origins of slow-waves in the insula, temporal, frontal, and parietal cortices. Approximately 46% of spontaneous slow waves across all subjects included at least one insula voxel within their origin (Murphy *et al* 2009, 2011). Sleep studies found the underlying active and inactive neuronal states occur locally in most slow-waves (Nir *et al* 2011). Our findings suggest that slow-waves during propofol anesthesia originate preferentially in the area around the anterior superior temporal gyrus. Since the insula is in the deeper areas around the anterior superior temporal gyrus, our results indicate that slow-waves might originate from the insula (supplementary figure 4). However, the underlying reason for this regional origin selectivity remains unknown. It might be that slow-waves originating in these areas could better propagate to other cortical areas, a hypothesis that is supported by the observation that the area around the lateral sulcus is important for initiating and propagating seizures in many cases of temporal lobe epilepsy (Isnard *et al* 2000, Ryvlin *et al* 2006).

Several studies indicate that slow-waves are not instantaneous events, but rather that they travel across the brain. Spontaneous slow-waves have distinct cortical origins with a characteristic propagation across the cortex, and involve unique subsets of cortical structures (Murphy *et al* 2009, 2011). For example, Murphy *et al* found one slow-wave propagation along the anterior–posterior axis that was mediated by a cingulate pathway, and another along the anterior–posterior axis over the lateral aspect of the cortex (Murphy *et al* 2009, 2011). In neurosurgical patients, Nir *et al* used simultaneous scalp EEG, intracerebral EEG, and unit firing from multiple brain regions to show that slow-waves can propagate from the medial prefrontal cortex to the medial temporal lobe and hippocampus (Nir *et al* 2011). Ruiz-Mejias *et al* found slow-wave propagation patterns in the anteroposterior axis in motor cortex and the mediolateral axis in visual cortex with multielectrode recordings in mice (Ruiz-Mejias *et al* 2011), and studies in cats found neural activity spread preferentially in the anterior-to-posterior direction (Volgushev *et al* 2006, Volgushev *et al* 2011). In our study, we found a spatiotemporal propagation along the anterior–posterior axis of the lateral aspect of the cortex (figure 3, supplementary figure 3), which is in general agreement with these studies.

Slow-wave activities are thought to reflect up and down states, and typically have a frequency below 1 Hz, largely between 0.2 and 0.5 Hz (Sanchez-Vives 2020). Extracting the instantaneous modulation frequency is crucial to understand when dynamic changes occur between brain states. However, slow-wave modulation is not purely sinusoidal, and methods that utilize narrow bandpass filters are not able to determine the modulation frequency at any given time-point (Purdon *et al* 2013, Mukamel *et al* 2014). This is one significant advantage of the tau-modulation method, which can extract the instantaneous modulation frequency (figures 4(A) and (B)), yielding more comprehensive insight into slow-wave modulation.

Anesthesia-induced slow-wave activity can modulate alpha oscillations at different polarities (peak-max or trough-max), depending on the depth of anesthesia (Purdon *et al* 2013, Mukamel *et al* 2014, Brown *et al* 2018), which most likely reflects underlying changes in the polarization level of the thalamus (Soplata *et al* 2017). The results of this study show that the tau-modulation method can reliably extract polarity from the TMC, providing significant insight into the state of unconsciousness (figures 4(C) and (D)).

Previous studies found a switch between the peak-max and trough-max slow-wave modulation before and after LOC (Purdon *et al* 2013, Stephen *et al* 2020). This effect was found to occur predominantly between slow-wave and alpha-band activity. A later study found trough-max slow-wave modulation to primarily occur within the low-frequency band (10–20 Hz) before LOC, and peak-max slow-wave modulation to occur within a broad frequency band after LOC (5–50 Hz) (Stephen *et al* 2020). This indicates that different underlying mechanisms might drive trough-max and peak-max slow-wave modulation. Our study mainly focuses on the modulation of slow-wave on broadband gamma activity, which is in general congruence with the results shown in Stephen *et al* (2020), where the trough-max effect did not show in higher frequencies (above 20 Hz) during the awake state.

One limitation of any method that calculates the coupling between amplitude and phase of a signal is that it may lead to more stochastic results in the absence of such coupling within the investigated frequency band. For example, the absence of a slow-wave during the awake state leads to unreliable results for the modulation frequency and polarity (see Figures 1(F)–(G). It is thus important to note, that for periods of low modulation strength, the modulation frequency and polarity may not be interpretable.

## 5. Conclusions

This study presents a novel tau-modulation method for slow-wave modulation analysis. This method extracts three important instantaneous modulation features (strength, frequency, and polarity) from a simple TMC, which can be derived from the cross-correlation between slow-wave and high-frequency signals. Combining the three modulation features in real-time provides a sensitive and physiologically-relevant marker of brain states related to unconsciousness, including depth of anesthesia, and may have applications in states of sleep and coma.

## Data Availability

The data that support the findings of this study are openly available at the following URL: http://neurotycho.org/anesthesia-task.

## Code availability

The MATLAB scripts necessary to reproduce the results presented in this manuscript are on GitHub https://github.com/neurotechcenter/TauModulation_SlowWave.
